# Thermodynamically Consistent Enthalpies of Adsorption of Mixtures from Classical Density Functional Theory

**DOI:** 10.1021/acs.jpcb.6c00492

**Published:** 2026-03-16

**Authors:** Philipp Rehner

**Affiliations:** † 27219 Energy and Process Systems Engineering, ETH Zurich, Zurich 8092, Switzerland; ‡ Molecular Engineering Thermodynamics, ETH Zurich, Zurich 8092, Switzerland

## Abstract

Classical density functional theory (cDFT) has been established as an efficient and robust framework for predicting adsorption isotherms. Moreover, the mathematical form of cDFT—an optimization instead of the more widely used molecular simulations—opens up additional opportunities based on calculating noise-free derivatives of interfacial properties. One of these opportunities is the rapid, consistent calculation of thermodynamic properties, such as the enthalpy of adsorption. This work showcases cDFT as a thermodynamically fully consistent model for fluids that describes all homogeneous and adsorbed phases with a single model, providing access to phase equilibria, density profiles, enthalpies, and more. Because the enthalpy of adsorption of a mixture is difficult to measure experimentally and is rarely discussed in modeling approaches, we first revisit its definition from an energy balance perspective and in the context of the Clausius–Clapeyron relation, independent of specific model assumptions. We follow this up by deriving expressions for the enthalpy of adsorption suitable for cDFT. The resulting framework is demonstrated using the PC-SAFT Helmholtz energy model for the adsorption of real gases in a model slit pore for a pure fluid, a binary mixture, and a multicomponent system.

## Graphical abstract

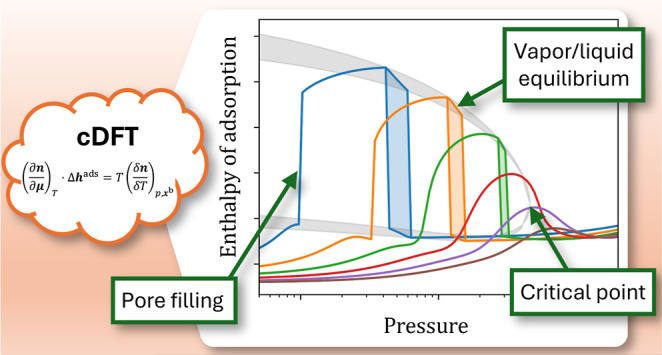

## Introduction

Adsorption technology plays a significant role in gas separation and storage applications, particularly for critical environmental challenges such as carbon capture,^1^ hydrogen purification,^2^ and natural gas storage.^3^ The lower energy costs compared to established thermal processes, such as distillation, make adsorption a promising technology for developing novel, energy-efficient, and sustainable processes in the energy transition.^4^ Predictive models for adsorption properties based on adsorbent structures can speed up the development process by determining optimal adsorbent material candidates early on in the design. Statistical mechanics provides a rigorous link between molecular structures and macroscopic measurable properties, such as adsorption isotherms. Most commonly, relations from statistical mechanics are evaluated in molecular simulations^5,6^ that generate samples from an ensemble—typically a grand canonical ensemble in adsorption—and average over the samples to obtain measurable quantities. Classical density functional theory (cDFT)^7,8^ provides an alternative to molecular simulation that reduces the computational effort by replacing sampling from an ensemble with determining the equilibrium distribution of atoms and molecules from a minimization of the grand potential functional. cDFT has been used to model gas adsorption for a variety of adsorbent materials, including zeolites,^9,10^ metal–organic frameworks (MOFs),^11–13^ covalent-organic frameworks (COFs),^14^ and porous carbons.^15,16^

While most studies using cDFT focus on the description of adsorption isotherms, caloric properties become relevant for determining heating and cooling requirements in adsorption process applications. In particular, the isosteric enthalpy of adsorption is related to the heat released when a gas component is adsorbed. Throughout the literature on the thermodynamic description of the enthalpy of adsorption, several controversies have persisted, including its sign, the definition of enthalpies of the adsorbed phase, and the relevance of properties such as spreading pressure and surface tension to the thermodynamics of porous materials.^17–22^

The application of cDFT to adsorption phenomena offers the opportunity to circumvent controversy by providing a consistent thermodynamic description of bulk and adsorbed phases that relies solely on the most basic and trusted thermodynamic principles. As a basis for the discussion, the remainder of this section reviews the enthalpy of adsorption in the context of adsorption process modeling and the Clausius–Clapeyron relation. The derived expressions are valid for any model describing the adsorbed phase and arbitrary porous material structures. Subsequently, cDFT is discussed as a method that can consistently describe homogeneous and adsorbed phases. We derive expressions for the direct evaluation of the enthalpy of adsorption from cDFT without numerical derivation of the Clausius–Clapeyron relation, and apply the method to the adsorption of condensable gases in a model slit pore.

## Enthalpy of Adsorption from Balance Equations

To demonstrate the relevance of the enthalpy of adsorption in engineering applications, this section derives the enthalpy of adsorption for mixtures from balance equations as they would be used, e.g., in a model of an adsorption process.

The adsorption system, as visualized in Figure 1, contains a porous material assumed here to be rigid. Therefore, the volume *V* of the system is always constant and not considered in the partial derivatives. The state of the fluid that is adsorbed in the system is defined by its temperature *T* and chemical potentials **μ** of all species. Throughout this manuscript, boldface refers to component-wise properties and a dot (·) denotes contraction over one index. The system exchanges heat δ*Q* and material δ*n* ^in/out^ with its surroundings. To be able to derive equilibrium properties like the enthalpy of adsorption, the state change of the system depicted in Figure 1 is assumed to be infinitely slow so that the system is constantly in an equilibrium state. Material is entering the system with an enthalpy *h* ^in^ and molar composition ***x*** ^in^ while material leaving the system is in equilibrium with the adsorbed phase, i.e., its enthalpy *h* ^b^ and composition *x* ^b^ are the ones of a bulk phase at *T* and **μ**. From these definitions, the energy balance

1 $$dU=h^{in}\delta n^{in}-h^{b}\delta n^{out}+\delta Q$$

and component balances

2 $$dn=x^{in}\delta n^{in}-x^{b}\delta n^{out}$$

follow. The internal energy *U* of the system contains contributions from the porous material and the adsorbed phase. Because we treat the adsorbent as rigid, the internal energy can be expressed as a function of temperature *T* and adsorbed amounts ***n***

3 $$dU={\left(\frac{\partial U}{\partial T}\right)}_{n}dT+{\left(\frac{\partial U}{\partial n}\right)}_{T}\cdot dn$$

Combining eqs 1 to 3 and solving for the exchanged heat δ*Q* leads to

4 $$\delta Q={\left(\frac{\partial U}{\partial T}\right)}_{n}dT+\left(x^{in}\cdot {\left(\frac{\partial U}{\partial n}\right)}_{T}-h^{in}\right)\delta n^{in}-\left(x^{b}\cdot {\left(\frac{\partial U}{\partial n}\right)}_{T}-h^{b}\right)\delta n^{out}$$

In eq 4, the heat capacity of the system (containing contributions from the porous material and the adsorbed fluid)

5 $$C_{V}={\left(\frac{\partial U}{\partial T}\right)}_{n}$$

and the molar enthalpy of adsorption

6 $$\Delta h^{ads}={\left(\frac{\partial U}{\partial n}\right)}_{T}-h^{b}$$

can be identified. Here the partial molar enthalpy ***h*** was used via *h* = ***x*** · ***h***. Eq 6 avoids the use of an enthalpy of the adsorbed phase, which falls apart in nanoporous materials or other microscopically inhomogeneous systems, where a scalar pressure is ill-defined. Importantly, that is not a choice made to avoid ambiguous definitions, but rather comes from the fact that a thermodynamic description of the adsorption process does not need those definitions in the first place. From eq 4 follows that a meaningful definition of the total molar enthalpy of adsorption Δ*h* ^ads^ is

7 $$\Delta h^{ads}=x^{b}\cdot \Delta h^{ads}=x^{b}\cdot {\left(\frac{\partial U}{\partial n}\right)}_{T}-h^{b}$$

because it can be used to simplify eq 4 to give

8 $$\delta Q=C_{V}dT+\left(h^{in}-h^{b}+\Delta h^{ads}\right)\cdot \delta n^{in}-\Delta h^{ads}\delta n^{out}$$

Eq 8 reinforces why it is important to distinguish between the enthalpy of adsorption and the heat of adsorption: using the definition of the heat released during isothermal adsorption (d*T* = 0, *T* ^in^ = *T*, δ*n* ^out^ = 0)

9 $$\delta Q_{T}=-q^{ads}\cdot \delta n^{in}$$

results in ***q*** ^ads^ = −Δ ***h*** ^ads^ for pure components and also ideal mixtures. However, real mixtures must account for the excess enthalpy difference between the inlet and equilibrium states. Even though the difference might be insignificant in most practical applications, the consequence is that the measured heat of adsorption depends on the composition of the inlet stream, which is defined by the process or experimental setting. The enthalpy of adsorption, on the other hand, is fully determined by the state *T* and **μ** or more conveniently *T*, *p* ^b^ and ***x*** ^b^ of the adsorbed fluid.

**Figure 1:**
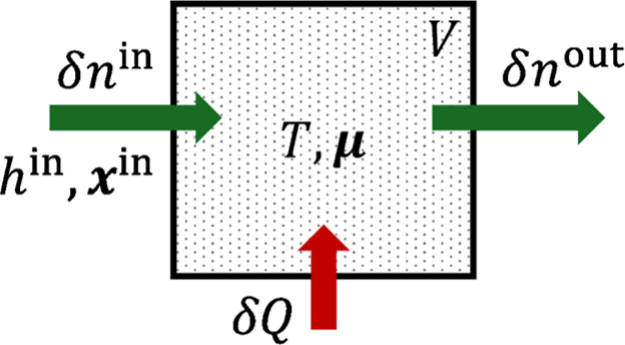
System boundary and flows of the basic adsorption process. In an equilibrium process, incoming and outgoing mass flows δ*n* ^in/out^ and heat crossing the system boundary δ*Q* are related via the component and energy balance, leading to the definition of the enthalpy of adsorption.

While the enthalpy of adsorption can be useful in process modeling to introduce effective simplifications to achieve computational tractability, it should also be noted that if a thermodynamically consistent model like cDFT is used to evaluate energy balances, it is much more efficient to not employ eq 3, but rather determine the internal energy of the adsorbed phase directly from the Helmholtz energy functional.

## The Clausius–Clapeyron Relation

In addition to adsorption process modeling, the enthalpy of adsorption plays a crucial role in temperature extrapolation of adsorption isotherms via the Clausius–Clapeyron relation. It is mostly used with the approximation of an ideal gas phase. Corrections for real gas behavior have been applied, but they are often based on specific isotherm models.^23–25^ Because it is difficult to find a general formulation that is applicable to mixture isotherms in the literature, the derivation is revisited here.

The Clausius–Clapeyron relation relates the *p*–*T* slope of a phase equilibrium to the enthalpy of phase change. It is most conveniently expressed in terms of the slope of the logarithmic pressure over inverse temperature

10 $$\frac{d\;\ln p}{d\frac{1}{RT}}=-\frac{RT^{2}}{p}{\left(\frac{\partial p}{\partial T}\right)}_{n}$$

The derivative is in the direction of the equilibrium. In the context of adsorption, and using the same system that is used in Figure 1, the derivative follows an isostere, i.e., a line with constant substance amount ***n***. The pressure *p* is related to the variables *T* and **μ** that define the inhomogeneous system by the Gibbs–Duhem relation

11 $$v^{b}dp=s^{b}dT+x^{b}\cdot d\mu$$

with the molar volume *v* ^b^ and the molar entropy *s* ^b^ of the bulk phase. Taking the derivative with respect to *T* at constant ***n*** of eq 11 and using it in eq 10 leads to

12 $$\frac{d\;\ln p}{d\frac{1}{RT}}=-\frac{RT^{2}}{pv^{b}}\left(s^{b}+x^{b}\cdot {\left(\frac{\partial \mu }{\partial T}\right)}_{n}\right)$$

The derivative of the chemical potentials **μ** can be replaced using a Maxwell relation based on the Helmholtz energy *F*

13 $${\left(\frac{\partial \mu }{\partial T}\right)}_{n}=\left(\frac{\partial^{2}F}{\partial T\partial n}\right)=-{\left(\frac{\partial S}{\partial n}\right)}_{T}$$

Using eq 13 in eq 12, together with the definition of the compressibility factor $Z^{b}=\frac{pv^{b}}{RT}$ , the molar enthalpy of a homogeneous phase *h* ^b^ = *Ts* ^b^ + ***x*** ^b^·**μ**, the fundamental equation for the internal energy at constant volume d*U* = *T*d*S* + **μ**·d ***n***, and finally the definition of the total molar enthalpy of adsorption eq 7, leads to the Clausius–Clapeyron relation for adsorption

14 $$\frac{d\;\ln p}{d\frac{1}{RT}}=-\frac{1}{Z^{b}}\left(h^{b}-x^{b}\cdot {\left(\frac{\partial U}{\partial n}\right)}_{T}\right)=\frac{\Delta h^{ads}}{Z^{b}}$$

Crucially, eq 14 is an exact thermodynamic relation that requires no assumptions beyond the rigidity of the adsorbent. At lower pressures (typical pressures for most gas adsorption processes), the compressibility factor of the gas phase *Z* ^b^ can be approximated by 1, which leads to the widely used simplified version of the Clausius–Clapeyron relation. However, the deviation from ideal gas behavior can become significant at higher pressures. Eq 14 is also reaffirming the definition of the enthalpy of adsorption in eq 7 that comes out of an energy balance of a porous medium: the same definition of multicomponent isosteric enthalpy of adsorption naturally reappears in the slope of an isostere via the Clausius–Clapeyron relation.

## Enthalpy of Adsorption from Classical Density Functional Theory

cDFT determines the density profile (and hence the adsorbed amounts ***n***) as a function of chemical potentials **μ** and temperature *T*. Therefore, it is convenient to rewrite the expression for the enthalpy of adsorption accordingly. Combining eqs 7, 12, and 14 and the partial molar entropy $s^{b}=-{\left(\frac{\partial \mu }{\partial T}\right)}_{p,x}$ relates the enthalpy of adsorption with partial derivatives of the chemical potential at constant adsorbed amounts ***n*** and at constant pressure *p* and bulk composition ***x*** ^b^

15 $$\Delta h^{ads}=T\left({\left(\frac{\partial \mu }{\partial T}\right)}_{p,x}-{\left(\frac{\partial \mu }{\partial T}\right)}_{n}\right)$$

The partial derivatives of the chemical potential can be replaced using the total differential of the adsorbed amount

16 $$dn={\left(\frac{\partial n}{\partial \mu }\right)}_{T}\cdot d\mu +{\left(\frac{\partial n}{\partial T}\right)}_{\mu }dT$$

which can be differentiated with respect to temperature at constant adsorbed amounts

17 $$0={\left(\frac{\partial n}{\partial \mu }\right)}_{T}\cdot {\left(\frac{\partial \mu }{\partial T}\right)}_{n}+{\left(\frac{\partial n}{\partial T}\right)}_{\mu }$$

and constant pressure and bulk composition

18 $${\left(\frac{\partial n}{\partial T}\right)}_{p,x^{b}}={\left(\frac{\partial n}{\partial \mu }\right)}_{T}\cdot {\left(\frac{\partial \mu }{\partial T}\right)}_{p,x^{b}}+{\left(\frac{\partial n}{\partial T}\right)}_{\mu }$$

Subtracting eq 17 from eq 18 leads to

19 $${\left(\frac{\partial n}{\partial \mu }\right)}_{T}\cdot \left({\left(\frac{\partial \mu }{\partial T}\right)}_{p,x^{b}}-{\left(\frac{\partial \mu }{\partial T}\right)}_{n}\right)={\left(\frac{\partial n}{\partial T}\right)}_{p,x^{b}}$$

which can be used with eq 15 to finally yield

20 $${\left(\frac{\partial n}{\partial \mu }\right)}_{T}\cdot \Delta h^{ads}=T{\left(\frac{\partial n}{\partial T}\right)}_{p,x^{b}}$$

Eq 20 is still valid for arbitrary adsorption models of the form ***n*** (*T*, **μ**). To apply it to cDFT, the derivatives of ***n*** = *∫* **ρ**(**r**)­d**r** are required.

### Implicit Differentiation of Density Profiles

In classical DFT, the equilibrium density profile in an open system is determined from a minimization of the grand potential functional Ω.

21 $$\min_{\rho \left(r\right)}\Omega \left(T,\mu ,\left[\rho \left(r\right)\right],\left[V^{ext}\left(r\right)\right]\right)$$

The grand potential is determined from the (intrinsic) Helmholtz energy functional *F*, the chemical potentials **μ** and the external potentials ***V*** ^ext^ via the Legendre transform

22 $$\Omega \left(T,\mu ,\left[\rho \left(r\right)\right],\left[V^{ext}\left(r\right)\right]\right)=F\left(T,\left[\rho \left(r\right)\right]\right)-\int \rho \left(r\right)\cdot \left(\mu -V^{ext}\left(r\right)\right)dr$$

Implicit differentiation can be used to determine the change in, or derivative of, the density profile with respect to temperature or chemical potential: Every solution of the unconstrained optimization problem, eq 21, fulfills the stationarity condition (the Euler–Lagrange equation)

23 $${\left(\frac{\delta \Omega }{\delta \rho \left(r\right)}\right)}_{T,\mu ,V^{ext}}=F_{\rho }\left(r\right)-\mu +V^{ext}\left(r\right)=0$$

Here and in the following, a shortened notation for derivatives of the Helmholtz energy in its canonical variables *T* and **ρ**(**r**) is used, e.g., $F_{\rho }\left(r\right)={\left(\frac{\delta F}{\delta \rho \left(r\right)}\right)}_{T}$ . Taking the derivative of eq 23, again assuming rigid solid structures, which implies d ***V*** ^ext^ = 0, leads to

24 $$F_{T\rho }\left(r\right)dT+\int F_{\rho \rho }\left(r,r^{\prime }\right)\cdot \delta \rho \left(r^{\prime }\right)dr^{\prime }-d\mu =0$$

which relates changes in densities δ**ρ**(**r**) to changes in temperature d*T* and chemical potentials d**μ**. From eq 24, the derivatives required for the enthalpy of adsorption in eq 20 can be determined, as

25 $$\int F_{\rho \rho }\left(r,r^{\prime }\right)\cdot {\left(\frac{\partial \rho \left(r^{\prime }\right)}{\partial \mu }\right)}_{T}dr^{\prime }=I$$

and

26 $$\int F_{\rho \rho }\left(r,r^{\prime }\right)\cdot {\left(\frac{\partial \rho \left(r^{\prime }\right)}{\partial T}\right)}_{p,x^{b}}dr^{\prime }=-s^{b}-F_{T\rho }\left(r\right)$$

Explicitly evaluating the Hessian *F*_**ρρ**_(**r**, **r**′) might be infeasible due to computational and especially memory limitations. Instead, eqs 25 and 26 can be solved efficiently using iterative solvers like GMRES that require evaluations of the Hessian-vector product *∫F*_**ρρ**_(**r**, **r**′)· ***v*** (**r**′)­d**r**′ for any given ***v*** (**r**) rather than the full Hessian. Using reverse automatic differentiation (backpropagation) on the Helmholtz energy functional not only significantly speeds up computations but also simplifies the implementation of the derivatives.^26^ From the derivatives of the density profile ${\left(\frac{\partial \rho \left(r\right)}{\partial \mu }\right)}_{T}$ and ${\left(\frac{\partial \rho \left(r\right)}{\partial T}\right)}_{p,x^{b}}$ , the derivatives of the adsorbed amounts ${\left(\frac{\partial n}{\partial \mu }\right)}_{T}$ and ${\left(\frac{\partial n}{\partial T}\right)}_{p,x^{b}}$ follow by integration over the unit cell.

### Case Study: Adsorption in a Slit Pore

The expressions for the enthalpy of adsorption presented in this work are valid for arbitrarily shaped (rigid) pores. We demonstrate the capability of cDFT to calculate enthalpies of adsorption for simple model slit pores, enabling all relevant conclusions to be drawn while keeping computational effort low. For an application of cDFT to gas adsorption in structured nanoporous materials like metal–organic frameworks (MOFs), including calculations of adsorption enthalpies, we refer to Dufour-Décieux et al.^13^ (pure components) and Thiele et al.^27^ (mixtures) who compare adsorption isotherms and enthalpies of gases in nanoporous materials to predictions from grand canonical Monte Carlo (GCMC) simulations. They show that for pure components and mixtures cDFT provides a multiple order of magnitude speed-up compared to GCMC simulations in exchange for an insignificant to moderate loss of accuracy depending on the shape and interaction strength of the gas molecules. The calculation of derivatives of density profiles and the enthalpy of adsorption is implemented in the FeOs software (v0.9.2)^28^ which is used in this study. The Jupyter notebook that reproduces all results and figures is available at https://gitlab.ethz.ch/epse/molecular-design-public/paper-enthalpy-of-adsorption. The computations, including postprocessing and data visualization, are completed within approximately 6 min on a single core of an AMD Ryzen 3975WX workstation CPU.

The external potential used to describe the model pore is given by

27 $$V_{i}^{ext}\left(z\right)=V_{i}^{LJ93}\left(\frac{L}{2}+z\right)+V_{i}^{LJ93}\left(\frac{L}{2}-z\right)$$

with the pore width *L* and the Lennard-Jones-9-3 potential^29^

28 $$V_{i}^{LJ93}\left(z\right)=\frac{2\pi }{45}m_{i}\varepsilon_{si}\sigma_{si}^{3}\rho_{s}\left(2{\left(\frac{\sigma_{si}}{z}\right)}^{9}-15{\left(\frac{\sigma_{si}}{z}\right)}^{3}\right)$$

The fluid/solid interaction parameters ε_si_ and σ_si_ are determined from Lorentz–Berthelot combining rules

29 $$\varepsilon_{si}=\sqrt{\varepsilon_{s}\varepsilon_{i}}\mathrm{and}\sigma_{si}=\frac{1}{2}\left(\sigma_{s}+\sigma_{i}\right)$$

The parameters describing the model slit pore used in this work are given in Table 1.

**Table 1:** Parameters Describing the Model Slit Pore

*σ*_s_ (Å)	*ε*_s_/*k*_B_ (K)	*ρ*_s_ (Å^–3^)	*L* (Å)
3.0	100.0	0.08	20.0

As shown in a previous study,^14^ the size parameter σ_*i*_ and the energy parameter ε_*i*_ from the PC-SAFT equation of state can be used to determine accurate fluid/solid interaction potentials, despite PC-SAFT not strictly modeling Lennard-Jones fluids. However, the elongation of the molecules needs to be accounted in eq 28 by multiplying the interaction potential with the chain length parameter *m*_*i*_. The same parameters are used to parametrize the PC-SAFT Helmholtz energy functional,^30^ which models fluid–fluid interactions in the cDFT frameworkbased on different intermolecular interactions

30 $$F^{PC-SAFT}=F^{ig}+F^{hs}+F^{hc}+F^{disp}+F^{assoc}$$

where “ig” refers to the ideal gas contribution, “hs” to the hard-sphere repulsion,^31–33^ “hc” to free-energy change due to chain formation,^34^ and “disp” to dispersive (van-der-Waals) attraction.^30^ The “assoc” term for associative interactions^35^ is used in systems that exhibit hydrogen bonds (here: the adsorption of pure methanol). The functional simplifies to the PC-SAFT equation of state^36^ for homogeneous density distributions and is therefore fully consistent with bulk properties and phase equilibria from PC-SAFT. A full list of pure-component PC-SAFT parameters for the molecules used in this work is shown in Table 2; the corresponding binary interaction parameters are shown in Table 3.

**Table 2:** Pure-Component PC-SAFT Parameters of the Molecules Used in This Work

component	*m*_*i*_ (−)	*σ*_*i*_ (Å)	ε_*i*_/*k*_B_ (K)	*n*_*i*_^A^ (−)	*n*_*i*_^B^ (−)	*κ*_*i*_^AB^ (−)	*ε*_*i*_^AB^/*k*_B_ (K)
methanol^37^	2.4858	2.7309	101.08	2	2	0.11953	1834.8
methane^38^	1.0000	3.7005	150.07				
ethane^38^	1.6069	3.5168	191.45				
propane^38^	1.9860	3.6244	209.09				
*n*-butane^38^	2.3112	3.7156	224.08				
carbon dioxide^38^	2.5310	2.5786	153.32				

**Table 3:** Binary Interaction Parameters *k*_*ij*_ for PC-SAFT Used in This Work^39^

	ethane	propane	*n*-butane	carbon dioxide
methane	–0.00489	–0.00551	0.00775	0.05976
ethane		0.01250	–0.00091	0.10596
propane			0.00277	0.12486
*n*-butane				0.13954

## Adsorption of a Pure Component

To facilitate the interpretation of results for multicomponent adsorption, we first demonstrate the thermodynamically consistent calculation of adsorption isotherms and enthalpies for a pure component.

Figure 2 shows the density profiles of methanol adsorbed in the model slit pore at 500 K together with the external potential defining the slit pore according to eq 27. At low pressures, methanol adsorbs at the pore walls. The relatively inconspicuous adsorption layer can be attributed to Lennard-Jones interactions between methanol molecules and the pore walls, which are weaker than the hydrogen bonds present in the adsorbed phase. Nevertheless, methanol is preferentially adsorbed in the pore, i.e., the pore-filling transition occurs at a pressure below the vapor pressure of the homogeneous phase.

**Figure 2:**
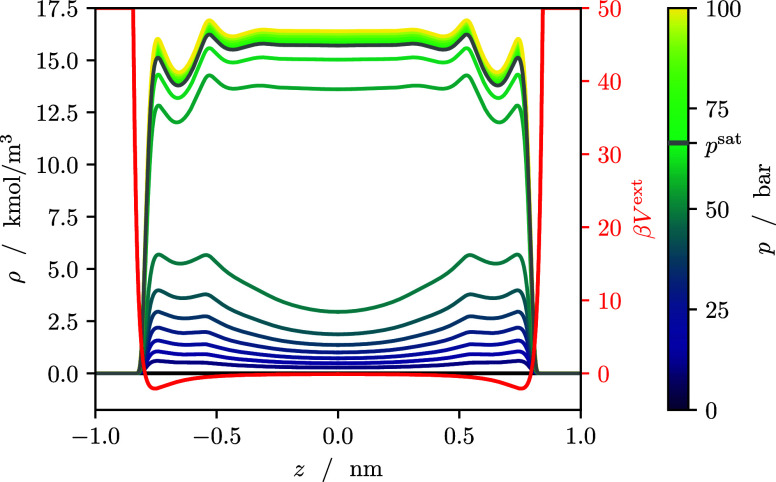
Density profiles of pure methanol adsorbed in a slit pore at 500 K and varying bulk pressures. In red: the external potential that defines the slit pore according to eq 27. In gray: the density profile corresponding to the bulk liquid/vapor coexistence at *p* ^sat^ = 66.4 bar.

This observation becomes clearer in Figure 3 (left), which shows the adsorption isotherms of methanol for temperatures between 430 and 580 K. In the following, adsorption isotherms are visualized in terms of the adsorption **Γ**, which is the total adsorbed amount *n* per surface area of the adsorbent. For the example slit pore, the adsorption is calculated as

31 $$\Gamma =\int_{0}^{L/2}\rho \left(z\right)dz$$

where the integration is over only one-half of the symmetric pore to account for each of the surfaces of the slit pore separately. At low temperatures, the isotherms exhibit a jump—the pore-filling transition—followed by a kink—the condensation of the bulk phase. Between 490 and 520 K, the jump in the loading transforms into a continuous increase, indicating a critical point of the pore-filling transition. At even higher temperatures, the isotherms surpass the critical temperature of the bulk methanol (528.07 K for this parametrization of PC-SAFT) and the shape of the isotherm becomes continuous and smooth as expected for supercritical gas adsorption.

**Figure 3:**
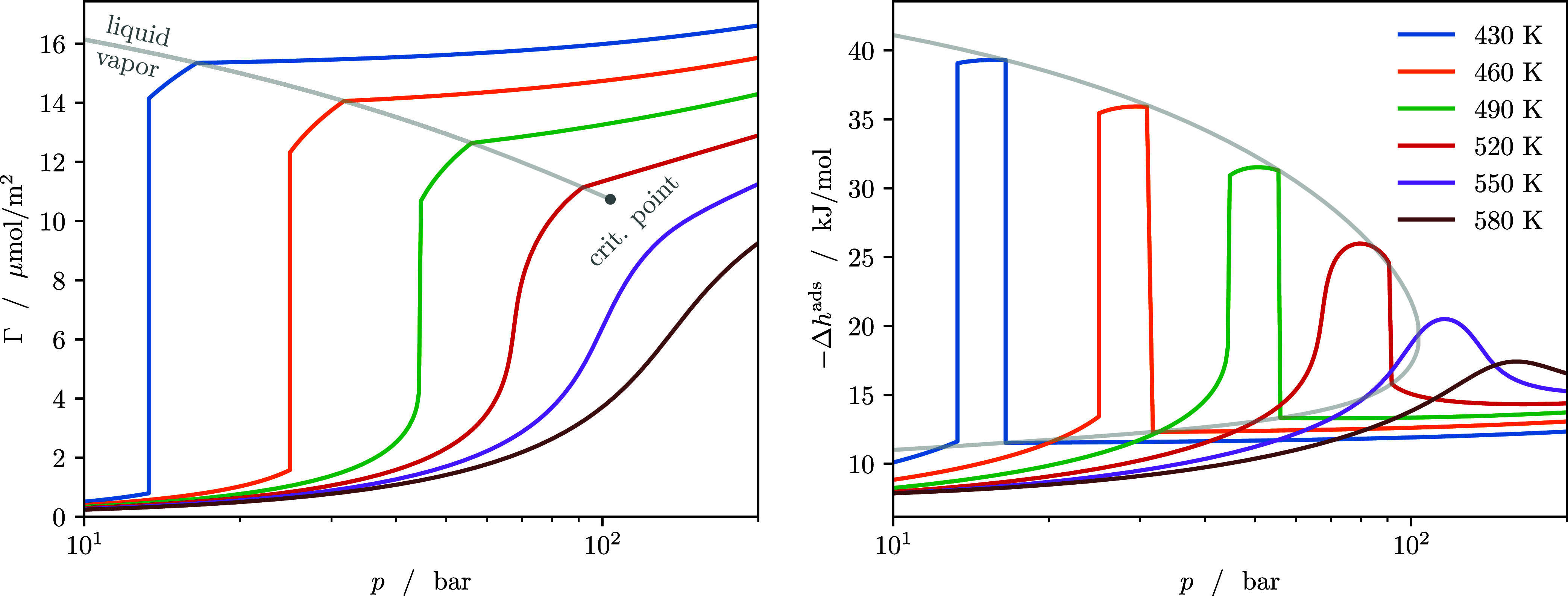
Adsorption isotherms (left) and enthalpy of adsorption (right) of methanol adsorbed in a slit pore at temperatures between 430 and 580 K. The gray line indicates the liquid/vapor phase transition in the bulk phase, i.e., the point along the isotherms at which the bulk phase is condensing.

The same changes in phase behavior can be observed from the enthalpies of adsorption shown in Figure 2 (right). At both low and high pressures, the enthalpy of adsorption is small. Only the state points for which pore condensation occurs, but the bulk phase is still in a vapor state, exhibit higher enthalpies of adsorption, with the peak becoming smaller toward and beyond the critical point of methanol. The derivative ${\left(\frac{\partial U}{\partial n}\right)}_{T}$ is unaffected by the condensation of the bulk phase. Therefore, from eq 7 follows that the jump in enthalpy of adsorption at the liquid/vapor phase transition (also indicated by the gray line in Figure 3 (right)) is precisely the enthalpy of vaporization of the bulk fluid. The significant increase in loading due to the pore-filling transition and the high enthalpy of adsorption associated with it is exploited in technologies such as adsorption heat pumps and chillers.^40–42^

### Binary Mixture

The adsorption isotherm and enthalpy of adsorption of a mixture at constant composition follow a similar trend to those of a pure component, with the added complexity of nonisothermal condensation in the bulk phase. Figure 4 shows the adsorption isotherm (left) and enthalpy of adsorption (right) of a binary, equimolar mixture of propane and *n*-butane in the same model split pore used throughout this study. Again, pore condensation occurs at pressures below the dew point line, as long as the temperature is below a certain critical temperature for the pore-filling transition (here between 350 and 400 K). At higher pressures, the isotherm bends twice—at the dew point and the bubble point. The nonisobaric condensation leads to a bifurcation in the enthalpy of adsorption: Depending on whether the equilibrium of the porous material with the gas phase or with the liquid phase is considered, the drop in adsorption enthalpy occurs either at the dew line or the bubble line.

**Figure 4:**
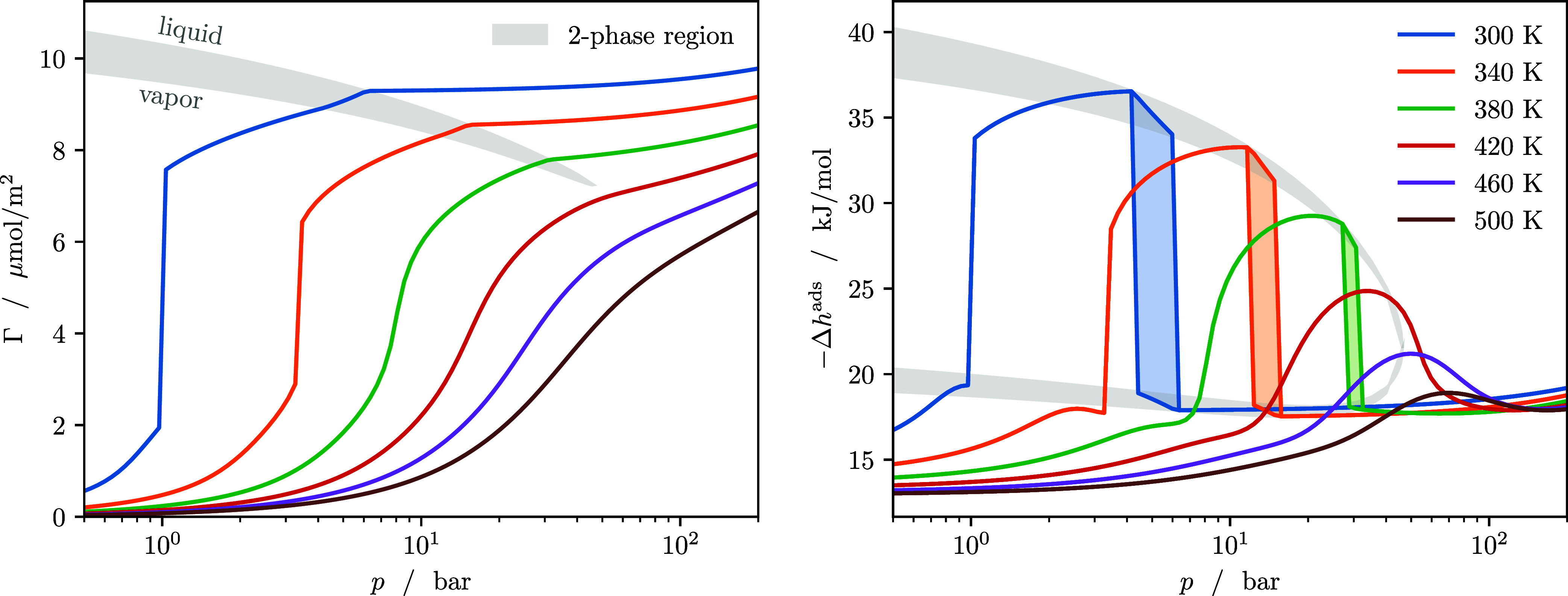
Adsorption isotherms (left) and enthalpy of adsorption (right) of an equimolar mixture of propane and *n*-butane adsorbed in a slit pore at temperatures between 300 and 500 K. The gray shaded area indicates the states in which the isotherms cross the 2-phase region of the corresponding bulk phase.

### Multicomponent Adsorption

Neither the expressions for the enthalpy of adsorption discussed in this work nor the cDFT framework limit the number of fluid components. Because the PC-SAFT Helmholtz energy is based on pair interactions, as with most molecular simulation methods, we can also assume that the model extrapolates robustly to systems with an arbitrary number of components. We conclude with an investigation of the adsorption of a model natural gas mixture containing methane, ethane, propane, *n*-butane, and carbon dioxide in the ratio 90:6:1.5:0.5:2.

Figure 5 shows the adsorption and the enthalpy of adsorption at *T* = 298 K. To better distinguish the trace components, the adsorbed amount is shown on a logarithmic scale. For low pressures (the Henry regime), the adsorption of each component is proportional to the pressure, because fluid/solid interactions dominate adsorption while fluid/fluid interactions are negligible. Therefore, longer alkanes are adsorbed with higher selectivity due to stronger interactions with the pore walls. The effect is visualized in Figure 6 that shows the density profiles of all species in the slit pore at three selected pressures. Again, the density is shown on a log scale to make the trace components more discernible. At 40 bar, the adsorption peak of *n*-butane and propane close to the wall is more than 2 orders of magnitude higher than the density in the bulk phase (dashed lines).

**Figure 5:**
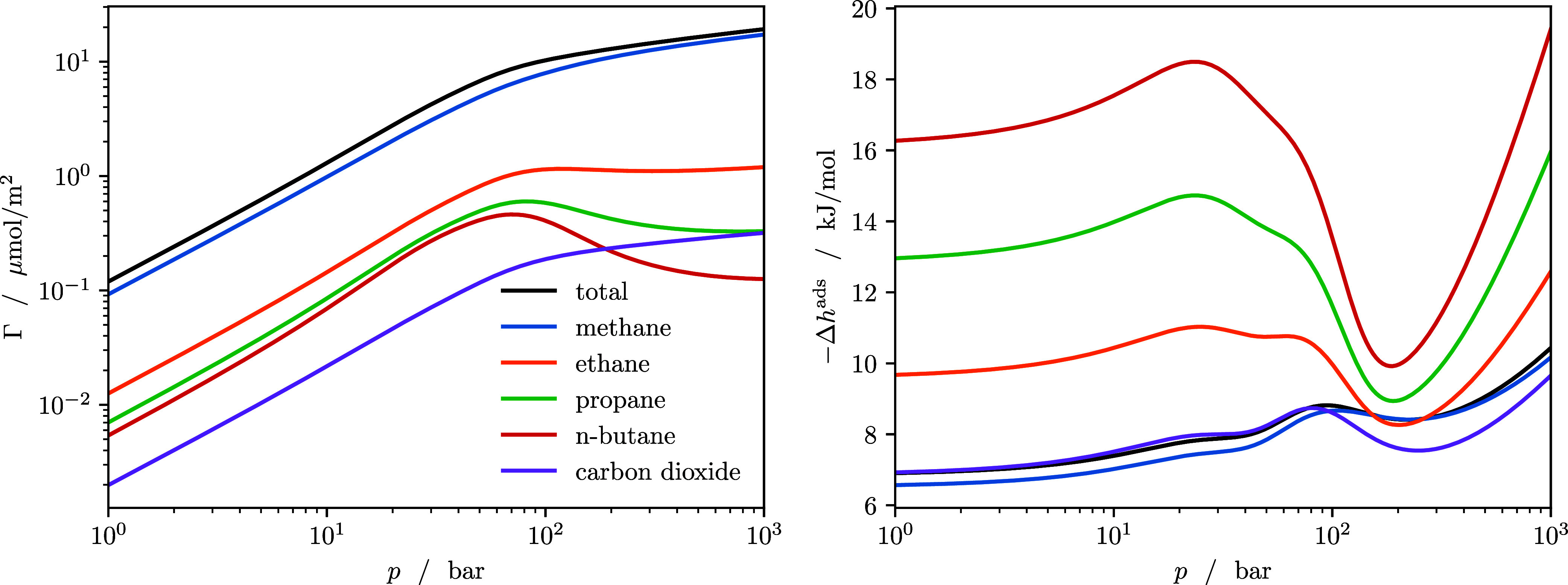
Adsorbed amount (left) and enthalpy of adsorption (right) of natural gas in a slit pore at *T* = 298 K. The adsorbed amounts and enthalpies of adsorption are shown for the individual components in the mixture and the total amounts.

**Figure 6:**
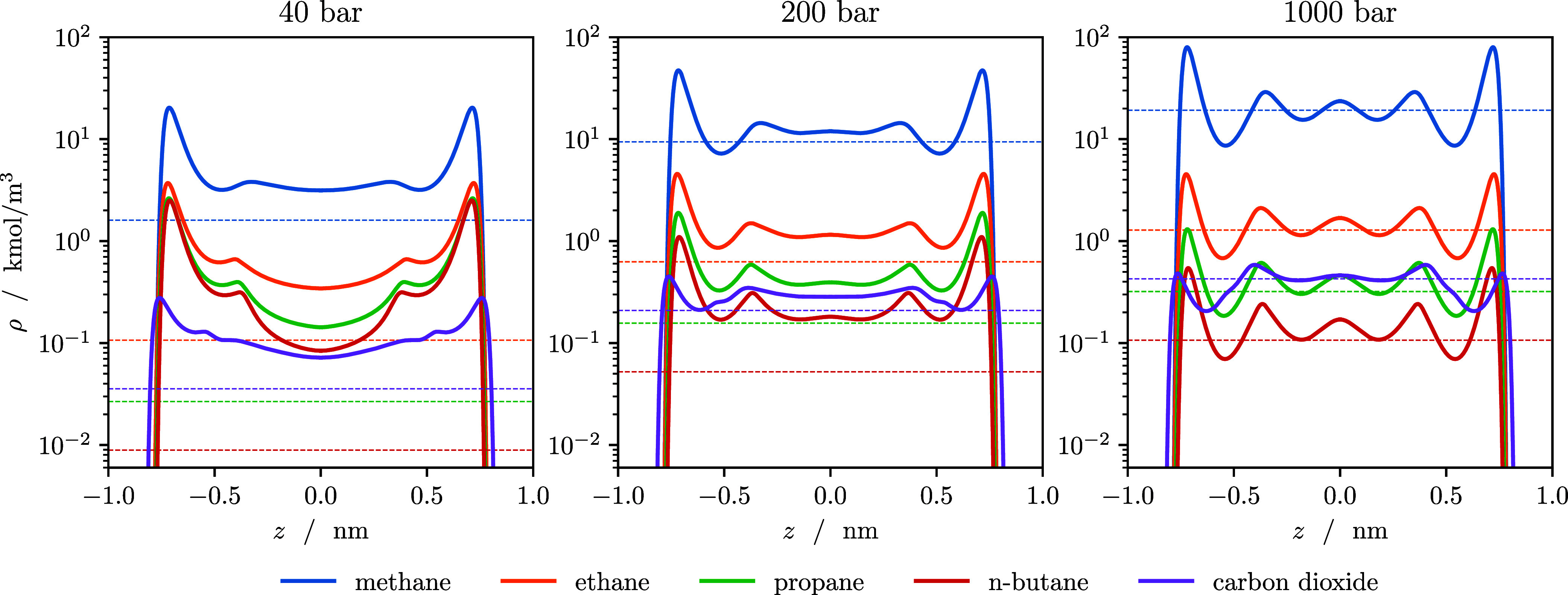
Density profiles of the natural gas mixture adsorbed in a slit pore at *T* = 298 K and three different pressures. The dashed line shows the corresponding bulk density of each component.

At higher pressures, the adsorbed amount of propane and *n*-butane undergoes a maximum, and the distribution of species in the pore converges more toward the composition of the bulk phase. In the same pressure region, the enthalpy of adsorption of the individual components dips, which leads to a significantly different shape of the enthalpy of adsorption over pressure compared to the pure-component enthalpies of adsorption in Figure 3. Thus, estimating enthalpies of adsorption for mixtures based on pure-component properties, e.g., via the ideal adsorbed solution assumption, can lead to erroneous predictions, which are mitigated when using a fully consistent framework for mixtures like cDFT.

## Conclusion

Classical density functional theory provides a thermodynamically fully consistent modeling framework that can describe equilibrium adsorption phenomena, such as adsorption isotherms and enthalpies. Because cDFT and GCMC share the same underlying statistical mechanics, the same properties can be evaluated from GCMC simulations, but at a significantly higher computational cost. Especially bulk properties, such as partial molar enthalpies, require significant additional computational effort in molecular simulations but come essentially for free with the cDFT approach.

On the other hand, the force fields used in molecular simulations set the standard for transferability and, therefore, predictive capability across a wide range of adsorbates. cDFT has been shown to reproduce GCMC results with satisfying accuracy for simple-shaped, weakly interacting gas molecules such as noble gases,^43^ small hydrocarbons,^14,44^ and hydrogen.^45^ For more polar or quadrupolar adsorbates (including nitrogen and carbon dioxide), deviations from molecular simulations can become large, depending on the adsorbent’s properties.^13^ Moreover, deviations in cDFT and GCMC from experimental data can be significant, posing a general and important challenge in the molecular modeling of adsorption phenomena.

Nevertheless, with further progress in the development of accurate Helmholtz energy models, cDFT proposes itself as a valuable tool to bridge molecular-scale phenomena with macroscopic properties of interfaces. Going from a simulation like GCMC to an optimization in cDFT removes stochastic noise and enables the calculation of derivatives. In this work, derivatives of density profiles in pores were used to determine the enthalpy of adsorption. However, the applications are manifold, like the calculation of derivatives with respect to model parameters for parameter optimization, with respect to structure descriptors for adsorbent design, or with respect to process variables for process design. cDFT can make molecular degrees of freedom directly accessible in the design of interfacially driven processes and, therefore, contribute to the development of novel, sustainable technologies.
